# Circular RNAs and their role in renal cell carcinoma: a current perspective

**DOI:** 10.1186/s12935-021-02181-7

**Published:** 2021-09-06

**Authors:** Zhongyuan Liu, Ming Li

**Affiliations:** Department of Urology, Shengjing Hospital of China Medical University, Shenyang, Liaoning 110004 People’s Republic of China

**Keywords:** Circular RNAs (circRNAs), Renal cell carcinoma (RCC), RCC promotor circRNAs, RCC suppressor circRNAs

## Abstract

Circular RNAs (circRNAs) are a new class of long non-coding RNAs, that results from a special type of alternative splicing referred to as back-splicing. They are widely distributed in eukaryotic cells and demonstrate tissue-specific expression patterns in humans. CircRNAs actively participate in various important biological activities like gene transcription, pre-mRNA splicing, translation, sponging miRNA and proteins, etc. With such diverse biological functions, circRNAs not only play a crucial role in normal human physiology, as well as in multiple diseases, including cancer. In this review, we summarized our current understanding of circRNAs and their role in renal cell carcinoma (RCC), the most common cancer of kidneys. Studies have shown that the expression level of several circRNAs are considerably varied in RCC samples and RCC cell lines suggesting the potential role of these circRNAs in RCC progression. Several circRNAs promote RCC development and progression mostly via the miRNA/target gene axis making them ideal candidates for novel anti-cancer therapy. Apart from these, there are a few circRNAs that are significantly downregulated in RCC and overexpression of these circRNAs leads to suppression of RCC growth. Differential expression patterns and novel functions of circRNAs in RCC suggest that circRNAs can be utilized as potential biomarkers and therapeutic targets for RCC therapy. However, our current understanding of the role of circRNA in RCC is still in its infancy and much comprehensive research is needed to achieve clinical translation of circRNAs as biomarkers and therapeutic targets in developing effective treatment options for RCC.

## Introduction

With trillions of cells multiplying in the body, any alterations in the process that lead to uncontrolled growth of cells will result in cancer. When tubular epithelial cells of nephron go cancerous, it gives rise to renal cell carcinoma (RCC) which accounts for over 90% of the renal malignancies, and over 3% of all adult malignancies [1]. The condition is mostly seen in old age (> 60 years), with nearly two times higher prevalence in males than in females [2]. Further, RCC is ranked as the sixth and eighth most common cancer in males and females respectively. Radical nephrectomy is the mainstay therapy for RCC, however distant metastasis and local invasion limits such an approach. In such cases, chemotherapy is an ideal choice but resistance to current drugs significantly impairs the treatment efficiency [3]. Therefore, novel strategies for early detection and targeted therapies are need of a moment for the successful management of RCC. A deeper understanding of the RCC pathophysiology may reveal relevant molecules for further advancement in the therapeutic management of RCC.

Similar to other cancers, the tumorigenesis of RCC involves dysregulation of genetic and epigenetic pathways [4–6]. In most RCC patients, the short arm of chromosome 3 is lost, where the tumor suppressor gene von Hippel-Lindau (VHL) is located, resulting in the dysregulation of the hypoxic pathway due to alteration of HIF-2α expression [7] Other pathways involved in cell proliferation and growth, like PI3K-AKT-mTOR pathways, are also activated in RCC [8]. Epigenetic disruption due to altered epigenetic regulators is identified as fundamental to cancer occurrence. Noncoding RNAs are one such epigenetic regulators that are shown to play a potential role in RCC development and progression [9–11]. Circular RNAs (circRNA) are a special category of long noncoding RNAs (lncRNAs) that are being extensively studied for their role in the development and progression of various types of cancer [12, 13]. Many studies have demonstrated that the expression of various circRNAs is dysregulated in various cancers including RCC [14–16]. Several circRNAs with enhanced expression in RCC models suggest the oncogenic potential of these overexpressed circRNAs in RCC [14]. On contrary, there are a few circRNAs that are downregulated in RCC demonstrating the tumor suppressor effect of circRNAs in RCC progression [14]. These studies demonstrate a critical role of circRNAs in various stages of RCC making them an important topic of research for developing new strategies to improvise RCC management. Hence, in the present review, we performed an extensive literature search for circRNAs associated with RCC and summarized the role of various circRNAs in RCC, demonstrating their potential to be used as biomarkers and targets for RCC therapy.

The overall structure of the article

Overview and biogenesis of circular RNAs.

Biological functions of CircRNAs.

Functional significance of CircRNAs in RCC.

circRNAs and their involvement in other cancer types.

**Fig. 1:**
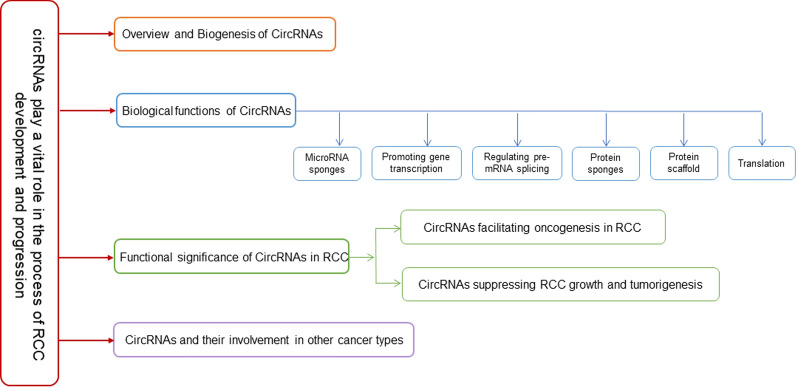
Flowchart of the research methodology

Conclusion

(Please see Fig. 1 for the flowchart of the research methodology).

### Overview and biogenesis of CircRNAs

**Fig. 2:**
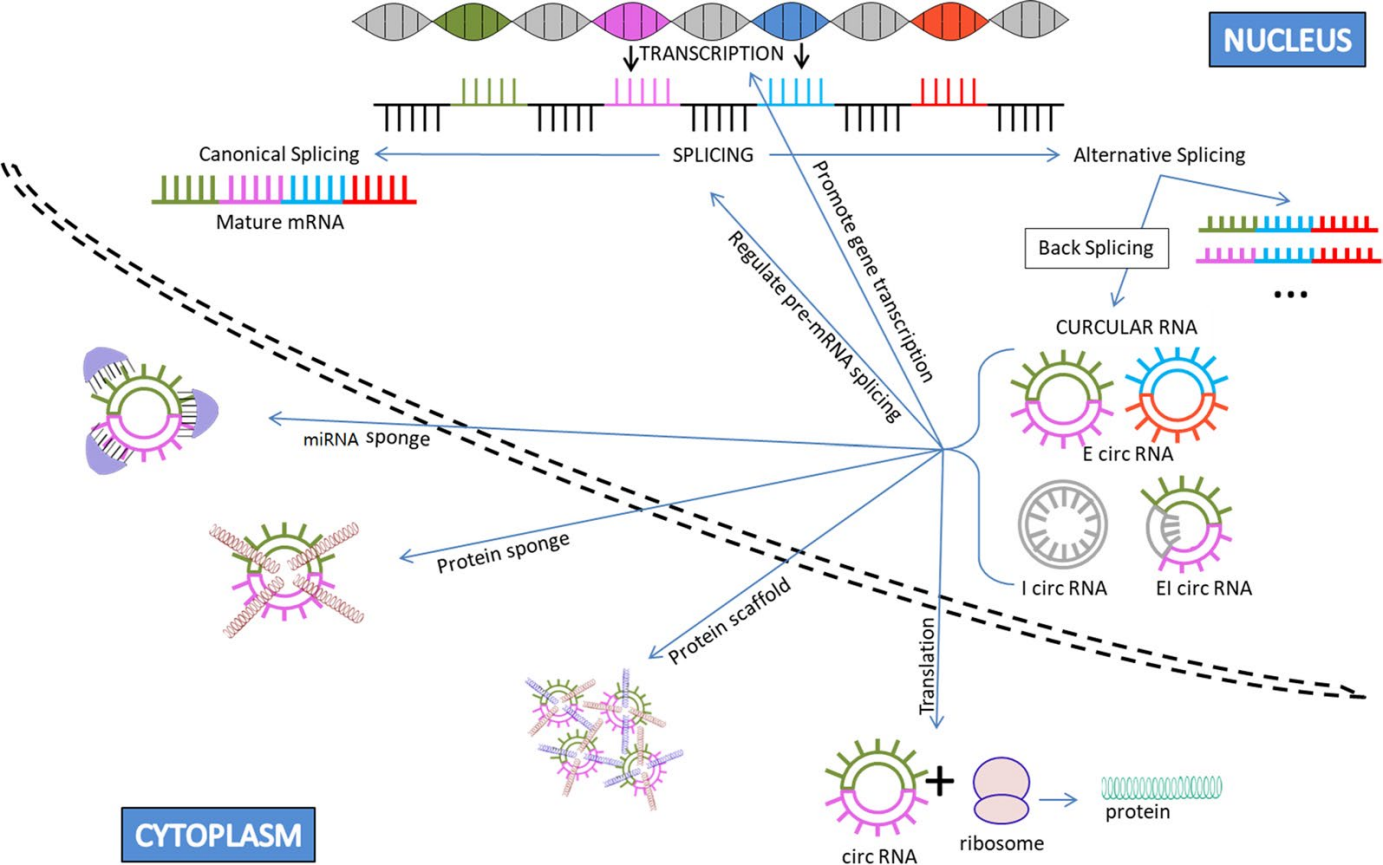
Biogenesis of circRNA and its biological function

CircRNAs are a subclass of lncRNAs that are generated by the process of back-splicing (Fig. 1), where a 5′ splice site is bonded to the 3′ splice site. Structurally, they are a single chain RNA molecule with a 5′–3′ phosphodiester bond, forming a covalently-looped circular structure [17]. Based on their composition, three types of circRNAs have been identified: exonic circRNAs (EcircRNAs), intronic circRNAs (IcircRNAs) and exon–intron circRNAs (EicircRNA) (Fig. 2) [18]. RNA binding proteins play a major role in the formation of circRNAs. For instance, Quaking supports EcircRNA formation by promoting a 5′–3′ phosphodiester bond formation [19], while muscleblind supports IcircRNA formation by binding to its own pre-mRNA [20]. Due to their unexpected structure, the finding of circRNAs was initially considered an artifact. However, with the use of latest technologies like next-generation sequencing and bioinformatics tools, the existence of circRNAs is not only accepted but over 30,000 types of circRNAs have been predicted to exist in humans [14, 21]. Important bioinformatics tools and databases useful for studying circRNAs are enlisted in Table 1. The expression of circRNAs is tissue specific and they are most abundantly found in neural tissue, where they tend to accumulate with age [22, 23]. The reason for this could be that the neurons exhibit highest rate of alternative splicing, an important process for circRNA biogenesis. Apart from that the circRNAs possess a longer half-life (18–23 h) compared to linear RAs (4–7 h) which could be one of the reasons they might get accumulated in hardly dividing cells like neurons and not much in highly proliferating tissue [24, 25]. The observed longer half-life in circRNA is due to the lack of 5′ and 3′ terminal structure making them relatively resistant to common RNA degradation pathways, and are thus considered stable of all the RNAs. Under in vitro conditions, the enzymatic activity of Rnase H and Rrp44 could cleave circRNAs, although the process is considerably slow [26, 27]. The mechanisms and rate of degradation of circRNAs in vivo are yet to be fully understood.

**Table 1:** List of important bioinformatics tools and databases useful for circRNA research

Tool/database name	Important features	Ref
Circ2Traits	It contains information of 1951 human circRNAs potentially associated with 105 different diseases. It also stores a putative miRNA-circRNA-mRNA-lncRNA interaction network for all these diseases	[28]
CircAtlas	It is a comprehensive database that contains 1070 RNA-seq samples from 6 different species with the integration of 1,007,087 circRNAs	[29]
CircBank	It is a human circRNA database that contains 12,348 conserved circRNAs and 4388 circRNAs with m6A modifications	[30]
Circbase	merged and unified data sets of circRNAs from multiple species	[31]
CIRCexplorer3	A comprehensive pipeline to quantitatively evaluate circRNA expression across samples	[32]
CircInteractome	A web-based tool for the analysis of circRNAs and their interacting proteins and miRNAs	[33]
CircNet	It provides tissue-specific circRNA expression profiles and circRNA-miRNA-gene regulatory networks	[34]
CIRCpedia v2	An updated database containing 180 RNA-seq datasets for circRNA annotations across 6 different species with computational tools to compare circRNA expression among different samples	[35]
CircPro	An integrated tool for the identification of circRNAs with protein-coding potential from high-throughput sequencing data	[36]
CircRNA disease	It provides a user-friendly interface for searching experimentally supported disease-associated circRNAs	[37]
CircRNADb	A comprehensive database comprising 32,914 non-redundant human exonic circRNAs with protein-encoding feature annotation	[38]
CircRNAFisher	A circRNA identification pipeline for robust circRNA identification	[39]
CIRI	A de novo circular RNA identification tool	[40]
CSCD	A comprehensive cancer-specific circRNA database	[41]
ExoRBase	It is a database containing 58,330 circRNAs, 15,501 lncRNAs and 18,333 mRNAs characterized from derived from RNA-seq data analyses of human blood exosomes	[42]
StarBase v2.0	A tool to identify the RNA–RNA and RNA–protein interactions including miRNAs, ncRNAs (lncRNAs, pseudogenes and circRNAs)	[43]

### Biological functions of CircRNAs

Despite their unusual structure and lack of 5′ and 3′ terminals, circRNAs are biologically active and are a hot topic of research. Various biological and cellular functions of circRNAs have been identified making them an important aspect of current biological research with respect to their role in various diseases such as cancer. Based on various studies, six biological functions of circRNAs have been identified and all the functions have been discussed briefly below. Moreover, all six functions of circRNAS have been represented in Fig. 2.

### Functioning as micro RNA (miRNA) sponges

Micro RNA (miRNA) is a type of non-coding RNA with a length of about 18–25 nucleotides, which erroneous expression has been confirmed to be related to cancer, autoimmune diseases, osteoporosis and so on [44–46]. CircRNAs can act as competitive endogenous RNAs, where they competitively bind to miRNAs via miRNA response elements and inhibit their functions [47]. For example, Zheng et al., found that the circHIPK3 can sponge 9 miRNAs, especially miR-124, which are known growth-suppressors in different cancer cells [48]. Similarly, circ-ITCH is shown to sponge miR-7 and miR-214 resulting in inhibition of lung cancer via increased expression of the ITCH gene [49]. Sponging miR-9 by circMTO1 increases p21 expression in hepatocellular carcinoma, resulting in the inhibition of its proliferation and invasive abilities [50]. Sponging by circRNA may not always result in inhibition of miRNA, but also serve as its reservoir or transporter. For example, the circRNA sponge for miR-7 (ciRS7) can sponge both miR-7 and miR-671, where the later could trigger the AGO2-mediated cleavage of ciRS7, releasing miR-7 [47, 51].

### Regulating transcription and translation

The circRNAs, primarily IcircRNAs and EicircRNAs, are able to influence gene transcription through their retained intronic sequences, by interacting with RNA polymerase II and U1 snRNP. For instance, studies have revealed that circEIF3J, circPAIP2, circANKRD52 and circSIRT7 could enhance the expression of their parental genes [52, 53]. Apart from regulation of gene transcription, the circRNA also influences the protein expression, mostly by acting as modulators of mRNA translation. For example, a study by Chao et al. showed that the circRNAs generated by the mouse formin (Fmn) gene prevented the translation of its mRNA into Fmn protein by harboring itself at its translation site [54].

### Competing with linear splicing of pre-mRNA

Both circRNA biogenesis and canonical splicing work on the same splice sites and depend on the same spliceosomal machinery, suggesting that the circRNAs compete with the linear splicing of pre-mRNAs [20, 55]. Studies have shown a negative correlation between circRNAs and their linear isoforms [56]. However, the molecular mechanisms underlying such competition needs to be further elucidated.

### Regulating translocation of various RNA binding proteins (RBPs)

CircRNAs can bind to RBPs and transport them to specific subcellular locations, and thus aid in regulating protein functions [57]. Studies have revealed that the RBPs like STAT3, PDK-1, AKT1 and c-myc are translocated into the nucleus by circ-Amotl1 [58–60]. While nuclear translocation of MBL protein and translational activator HuR are facilitated by circ-Mbl and circPABPN1, respectively [20, 61].

### Acting as a scaffold for protein interaction

By acting as scaffolds, some circRNAs are shown to influence the kinetics of the protein–protein interaction by facilitating the contact between them. For instance, circ-Amotl1 acts as a scaffold for PDK1 mediated AKT1 phosphorylation that aids in its nuclear translocation [62], while circ-Foxo3 can facilitate the interaction between p53 and Mdm2 that results in degradation of p53 [63].

### Encoding for peptides and proteins via translation

Owing to its unusual structure, circRNAs were initially thought to be untranslatable. However, recently several studies reported that circRNAs also get encoded [64–66]. Despite the absence of 5′ and 3′ terminals, the circRNA demonstrates a cap-independent open reading frame that incorporates internal ribosome entry sites, allowing its translation via membrane-associated ribosomes [64]. Yang et al., showed that in the presence of N^6^-methyladenosine, some circRNAs within in cancer cell line could encode several peptides [66]. Legnini et al. also demonstrated the presence of heavy polysomes in circ-ZNF609, which can be translated into a protein that may control myoblast proliferation [65]. More studies are needed in the sector to not only reveal the proteins coded by circRNAs but also to elucidate their functional relevance.

### Functional significance of CircRNAs in RCC

**Fig. 3:**
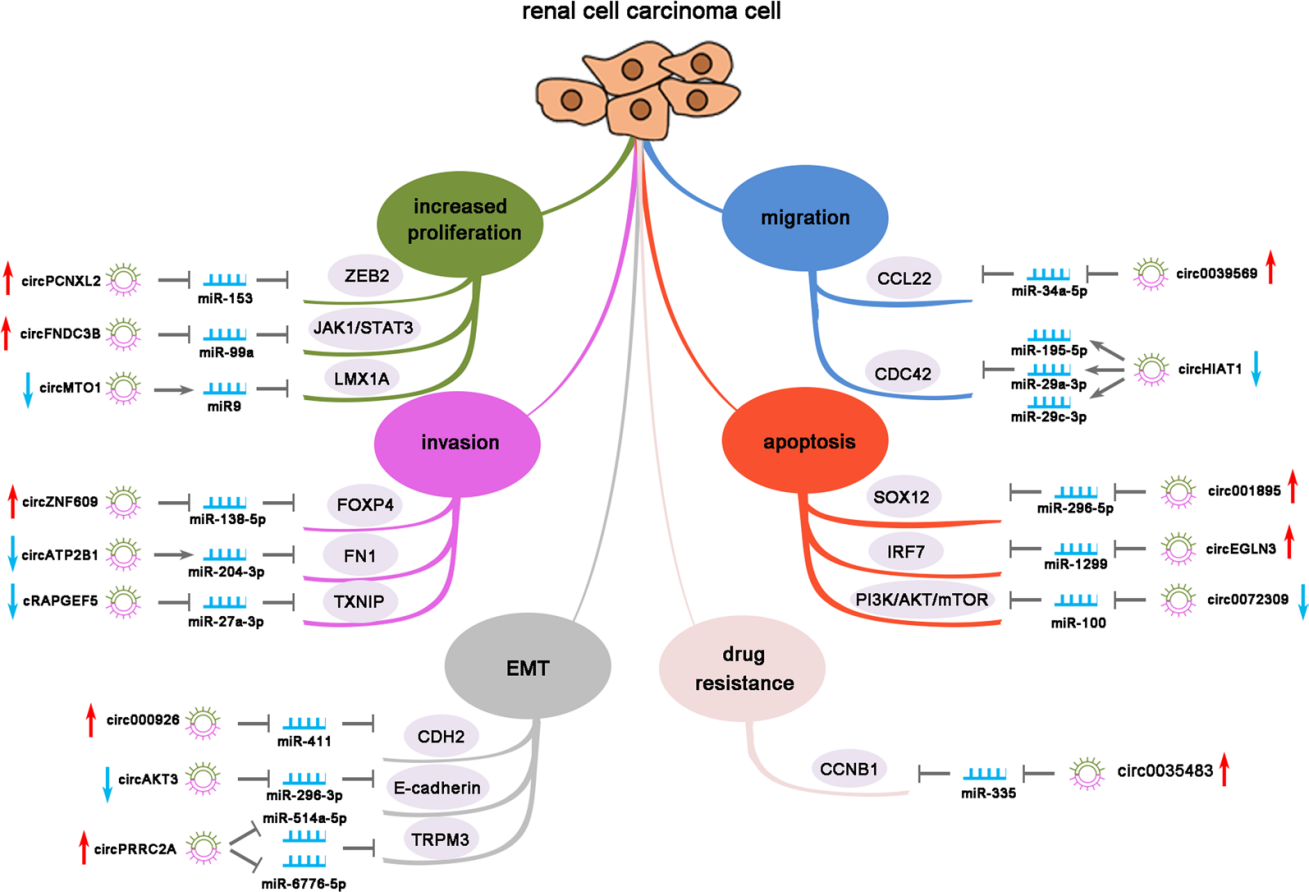
Circular RNA affects the tumorigenesis mechanism of renal cell carcinoma

Owing to such diverse biological activities, circRNAs play a critical role in human physiology and pathology, including cancers. Here we discuss the role of circRNAs in RCC with emphasis on oncogenic and tumor suppressor functions of various circRNAs. The mechanisms of how the circular RNAs impact the tumorigenesis of renal cell carcinoma are shown in Fig. 3. Several genome-wide studies have reported altered transcriptional profiles of circRNA in RCC. Franz et al. identified 13,261 circRNAs in clear cell renal cell carcinoma (ccRCC) samples, of which 78 were upregulated and 91 were downregulated as compared to matched controls [67]. The bioinformatics analysis of the RNA microarray database of ccRCC tissues by Ma et al. revealed that the expression of a total of 542 circRNAs was deviated from normal, among which 218 circRNAs were upregulated while the remaining 324 were downregulated [68]. Mechanism of many of these circRNAs in the regulation of key processes of RCC tumorigenesis, like epithelial–mesenchymal transition (EMT), proliferation, migration, invasion, apoptosis and drug resistance have been identified [14, 69–72]. Each upregulated or downregulated circRNA can influence multiple key processes via circRNA/miRNA/miRNA-target gene axis, the details of which are discussed briefly in Tables 2 and 3.

**Table 2:** CircRNAs with oncogenic functions in RCC

CircRNA	Target miRNA/gene axis	Gene/Protein activity	Functions in RCC	Ref
circPCNXL2	miR-153/ZEB2	Increased	Increased proliferation and invasion	[73]
circ0039569	miR-34a-5p/CCL22	Increased	Increased proliferation, migration and invasion	[72]
circZNF609	miR-138-5p/FOXP4	Increased	Increased proliferation and invasion	[75]
circFNDC3B	miR-99a/JAK1/STAT3/MEK/ERK	Increased	Increased proliferation and migration	[76]
circNRIP1	miR-505/AMPK/PI3K/AKT/mTOR	Increased	Increased proliferation and migration	[77]
circ001895	miR-296-5p/SOX12	Increased	Increased proliferation, migration, invasion and decreased apoptosis	[78]
circEGLN3	miR-1299/IRF7	Increased	Increased proliferation, migration, invasion and decreased apoptosis	[79]
circABCB10	–	–	Increased proliferation and migration decreased apoptosis	[80]
circ000926	miR-411/CDH2	Increased	Increased proliferation, migration, invasion and EMT	[85]
circPRRC2A	miR-514a-5p miR-6776-5p/TRPM3	Increased	Increased proliferation and EMT	[69]
circ0035483	miR-335/CCNB1	Increased	Increased proliferation, autophagy and resistance to Gemcitabine	[70]

**Table 3:** CircRNAs with tumor suppressive functions in RCC

CircRNA	Target miRNA/gene axis	Gene/protein activity	Functions in RCC	Ref
hsa-circ0072309	miR-100/PI3K/AKT/mTOR	Increased	Increased proliferation, migration and invasion; decreased apoptosis	[86]
circ0001451	–	–	Increased proliferation; decreased apoptosis	[81]
circAKT3	miR-296-3p/E-cadherin	Decreased	Increased migration, invasion and EMT	[87]
cRAPGEF5	miR-27a-3p/TXNIP	Decreased	Increased proliferation,migration and invasion	[71]
circATP2B1	miR-204-3p/FN1	Increased	Increased migration and invasion	[88]
circHIAT1	miR-195-5p miR-29a-3p miR-29c-3p/CDC42	Increased	Increased migration and invasion	[89]
circMTO1	miR9/LMX1A	Decreased	Increased proliferation and invasion	[90]

### CircRNAs facilitating oncogenesis in RCC

Several circRNAs are found upregulated in RCC which is correlated with tumor growth. RNA microarray analysis by Zhou et al. found the upregulation of circPCNXL2 in ccRCC samples, that correlated with poor overall survival of the patients [73]. Knockdown of circPCNXL2 resulted in decreased proliferation and invasion of RCC cells in vitro and significantly reduced the tumor growth in vivo [73]. Further experimental analysis showed that circPCNXL2 functions as a miRNA sponge for miR-153, resulting in increased expression of ZEB2 protein, which is associated with aggressive RCC phenotype and poor prognosis in RCC patients [73, 74]. Similarly, Jin et al. determined an oncogenic role of circ0039569, where it could support the survival and metastasis of RCC by promoting the proliferation, invasion and migration of RCC cells [72]. Circ0039569 was found to achieve this by sponging miR-34a-5p that resulted in upregulation of CCL22 gene, which codes for CCL22 chemokine [72]. CircZNF609 is another oncogenic circRNA which is shown to promote the proliferation and invasion of RCC cells [75]. RNA immunoprecipitation assay and luciferase assay revealed the role of circZNF609/miR-138-5p/FOXP4 axis in RCC tumorigenesis [75]. CircFNDC3B and circNRIP1 are two other circRNAs that are shown to promote the proliferation and migration in RCC cells. CircFNDC3B negatively regulates miR-99a influencing the JAK1/STAT3 and MEK/ERK pathway resulting in increased proliferation and migration of RCC cells [76]. Similarly, circNRIP1 played the oncogenic role in RCC via miR-505/AMPK and miR-505/PI3K/AKT/mTOR pathway [77].

Few circRNAs could impart anti-apoptotic features to RCC cells. Increased SOX12 expression due to sponging of miR-296-5p by circ001895 could not only increase proliferation, invasion and migration of RCC cells but also prevented their apoptosis by increasing Bcl-2 and decreasing Bax and cleaved caspase-3 expression [78]. Moreover, apoptosis of RCC cells was prevented by circEGLN3 via miR-1299/IRF7 [79], and circABCB10 [80], by altering Bax, Bcl2 and caspase-3 protein expression. CircEGLN3, along with circNOX4 and circRHOBTB3 were all found to correlate well, demonstrated by good area under the receiver operating characteristic curve (AUC-ROC), with the clinical features and overall survival of ccRCC patients, indicating their potential as diagnostic biomarkers for the condition [67, 81]. Of these, circEGLN3 demonstrated an excellent AUC-ROC of 0.98, making it a remarkably reliable biomarker [79]. CircABCB10 was found to be associated well with the pathologic grade and TNM staging of RCC and thus may serve as a potential prognostic marker [80].

Epithelial–mesenchymal transition (EMT) is a critical process through which the differentiated epithelial cells acquire the features of stem-like mesenchymal cells, contributing to tumorigenesis, cardiopathy and other diseases [82–84]. EMT is a major dysregulated element in RCC, which is reportedly promoted by circ000926 and circPRRC2A via miR-411/CDH2 and miR-514a-5p/miR-6776-5p/TRPM3 axis [69, 85]. Further, circPRRC2A level correlated well with the tumor size, Fuhrman grade and pT stage which makes it an independent prognostic biomarker for overall survival and metastasis-free survival [85]. Yan et al. reported the role of circ0035483 in contributing to gemcitabine resistance in human RCC cells by targeting miR-335/CCNB1 axis [70]. Together, upregulation of these circRNAs contributes to the tumorigenesis and progression of RCC.

### CircRNAs suppressing RCC growth and tumorigenesis

Following the same circRNA/miRNA/target gene axis, several circRNAs can exert anti-tumor effects in RCC. Chen T et al. identified that the Hsa-circ0072309 was poorly expressed in RCC specimens [86]. Increasing the expression of Hsa-circ0072309 in RCC cell lines, in which the expression was otherwise suppressed, inhibited their proliferation, migration and invasion abilities, while enhancing their apoptosis. The search for underlying mechanisms revealed the sponging of miR-100 by Hsa-circ0072309 that led to suppression of PI3K/AKT/mTOR pathway in RCC cells [86]. Another circRNA that could induce apoptosis in RCC cells is circ0001451 and knockdown of which resulted in a significant RCC growth under in vitro conditions [81]. Further, an AUC-ROC of 0.704 circ0001451 was found to be correlated well with the clinicopathological features and overall survival of ccRCC patients, making it an attractive diagnostic and prognostic biomarker [81]. By regulating miR-296-3p/E-cadherin axis, circ-AKT3 was shown to inhibit EMT in RCC cells resulting in suppression of metastasis of ccRCC [87]. CircRNAs cRAPGEF5 and circATP2B1 are also shown to suppress the proliferation, migration and invasion of RCC cells by regulating the miR-271-3p/TXNIP and miR-204-3p/FN1 axis, respectively [71, 88]. Further, the expression level of circRAPGEF5 correlated well with tumor stages, overall survival and relapse-free survival of RCC patients and thus may serve as a prognostic biomarker in RCC [71]. circHIAT1 can sponge multiple RNAs that include miR-195-5p, miR-29a-3p and miR-29c-3p to suppress CD-42 expression, which leads to suppression of migration and invasion in ccRCC cells [89]. Another circRNA with an anti-tumor function in RCC is circMTO1, which promotes the expression of tumor suppressor LMX1A by acting as a miR9 sponge and leading to miR9 downregulation. LMX1A is a direct target of miR9 and downregulation of miR9 by circMTO1 leads to higher expression of LMX1A and ultimately leading to suppression of RCC progression demonstrating the role of circMTO1 as a potential therapeutic target for RCC therapy [90]. All these circRNAs with tumor suppressor functions are downregulated in RCC, and the fact that their upregulation can inhibit the RCC progression, metastasis and chemoresistance under in vitro conditions, makes them potential therapeutic targets for treating RCC. Many more circRNAs undoubtedly play a crucial role in RCC tumorigenesis which are yet to be discovered by future research.

### CircRNAs and their involvement in other cancer types

Compared with other published articles, we have made a more comprehensive and novel summary of the role of circRNAs in RCC. At the same time, it is not only limited to the role of circRNAs in RCC, we also summarize the reports of circRNAs in other cancers. Different circRNAs and their involvement in various cancer types are summarized in Table 4. According to the information summarized in the table, various studies on different circRNAs suggest that they play an important role not only in RCC but in other types of cancers also including breast cancer, colorectal cancer, gastric cancer, hepatocellular carcinoma, glioma, lung cancer, bladder cancer and hematological malignancies [91–93]. In these cancer types, differential expression of various circRNAs has been reported suggesting their crucial role in cancer development and progression. Generally, circRNAs with enhanced expression in different cancer types play an oncogenic role via targeting the expression of important miRNAs and proteins leading to tumorigenesis [91, 92]. On the other hand, circRNAs with diminished expression in different cancer types act as a tumor suppressor and when ectopically expressed leads to suppression of tumor growth [91, 92].

**Table 4:** List of important circRNAs associated with cancers other than RCC

Sr. No.	CircRNA	Cancer types	Expression pattern	Proposed function	Ref
1	circFBXW7	Glioblastoma	↓	Tumor suppressor	[94]
2	circITCH	Glioma	↓	Tumor suppressor	[95]
		Multiple myeloma	↓	Tumor suppressor	[96]
		Bladder cancer	↓	Tumor suppressor	[97]
		Cervical cancer	↓	Tumor suppressor	[98]
		Breast cancer	↓	Tumor suppressor	[99]
		Osteosarcoma	↓	Tumor suppressor	[100]
		Ovarian cancer	↓	Tumor suppressor	[101]
		Hepatocellular carcinoma	↓	Tumor suppressor	[102]
3	circSMARCA5	Glioblastoma	↓	Tumor suppressor	[103]
		Hepatocellular carcinoma	↓	Tumor suppressor	[104]
		Non-small cell lung cancer	↓	Tumor suppressor	[105]
4	circSHPRH	Glioma	↓	Tumor suppressor	[106]
5	circZKSCAN1	Hepatocellular carcinoma	↓	Tumor suppressor	[107]
6	circSLC8A1	Bladder cancer	↓	Tumor suppressor	[108]
7	circ-ZFR	Gastric cancer	↓	Tumor suppressor	[109]
8	circPTK2 (hsa_circ_0008305)	Non-small cell lung cancer	↓	Tumor suppressor	[110]
9	circSMAD2	Hepatocellular carcinoma	↓	Tumor suppressor	[111]
10	circ_0132266	Chronic lymphocytic leukemia	↓	Tumor suppressor	[112]
11	circ_0000190	Multiple myeloma	↓	Tumor suppressor	[113]
12	CDR1as	Non-small cell lung cancer	↑	Oncogenic	[114]
		Colorectal cancer	↑	Oncogenic	[115]
		Hepatocellular carcinoma	↑	Oncogenic	[116]
13	circHIPK3	Colorectal cancer	↑	Oncogenic	[117]
		Gallbladder cancer	↑	Oncogenic	[118]
		Chronic myeloid leukemia	↑	Oncogenic	[119]
14	circNFIX	Glioma	↑	Oncogenic	[120]
		Non-small cell lung cancer	↑	Oncogenic	[121]
		Pituitary adenoma	↑	Oncogenic	[122]
15	circNT5E	Glioblastoma	↑	Oncogenic	[123]
		Non-small cell lung cancer	↑	Oncogenic	[124]
		Bladder cancer	↑	Oncogenic	[125]
16	circTTBK2	Glioma	↑	Oncogenic	[126]
17	hsa_circ_0046701	Glioma	↑	Oncogenic	[127]
18	circ100284	Osteosarcoma	↑	Oncogenic	[128]
19	circ-DNMT1	Breast cancer	↑	Oncogenic	[129]
20	circ-BANP	Colorectal cancer	↑	Oncogenic	[130]
		Lung cancer	↑	Oncogenic	[131]
21	circRNA_001569	Gastric cancer	↑	Oncogenic	[132]
		Colorectal cancer	↑	Oncogenic	[133]
		Breast cancer	↑	Oncogenic	[134]
		Pancreatic cancer	↑	Oncogenic	[135]
		Osteosarcoma	↑	Oncogenic	[136]
		Hepatocellular carcinoma	↑	Oncogenic	[137]
		Non-small cell lung cancer	↑	Oncogenic	[138]
22	circPAN3	Acute myeloid leukemia	↑	Oncogenic	[139]
23	circ_0007841	Multiple myeloma Ovarian cancer	↑	Oncogenic	[140]
			↑	Oncogenic	[141]
24	circFGFR1	Non-small cell lung cancer	↑	Oncogenic	[142]
25	circTP63	Lung squamous cell carcinoma	↑	Oncogenic	[143]
		Breast cancer	↑	Oncogenic	[144]
		Hepatocellular carcinoma	↑	Oncogenic	[145]
26	circNRIP1	Gastric cancer	↑	Oncogenic	[146]
		Cervical cancer	↑	Oncogenic	[147]
		Ovarian cancer	↑	Oncogenic	[148]
		Nasopharyngeal carcinoma	↑	Oncogenic	[149]
		Osteosarcoma	↑	Oncogenic	[150]

↓—down-regulation, ↑—up-regulation

## Conclusion and future perspective

Circular RNAs are a group of biologically active long non-coding RNAs that are associated with various biological functions in eukaryotic cells. Although initially neglected as an artifact, advancement in recent research has led to a better understanding of their functions and applicability, particularly in the field of cancer. Here we have demonstrated that there is significant dysregulation in the expression of various circRNAs in cancers including RCC. We further gave an overall idea of how several circRNAs influence RCC growth and progression. We further provided detailed examples and a comprehensive list of circRNAs with oncogenic and tumor suppressive effects in RCC, demonstrating the role of various circRNAs in the complex process of RCC development. However, our knowledge of the role of circRNAs in cancers including RCC is very limited and there is still a need for more research related to circRNAs to determine their internal structure and entire functional spectrum in cancer biology. Our understanding of the role of circRNA in RCC is in its infancy, as only a few circRNAs have been identified. Even within the identified circRNAs, most of our current understanding of their mechanism of action is limited to miRNA sponging activity, while much of their other functions are yet to be understood. The tumor microenvironment (TME) is a complex ecosystem that plays a vital role in the process of RCC development and progression. However, the role of circRNA in shaping the RCC TME and vice versa is still elusive [151]. CircRNAs are abundantly expressed in body fluids [152–154], and the fact that they possess a longer half-life and better stability make them attractive biomarkers in liquid biopsy for diagnosis or monitoring of various conditions including RCC. However, not all the current known circRNAs associated with RCC can serve as biomarkers, and those that are considered as potential diagnostic or prognostic biomarkers need more studies to establish their credibility. In fact, the techniques and methods to reliably detect circRNAs need to be further standardized. Within our current understanding, circRNAs seem to be promising agents for targeted therapy, however, we are far from determining the methods to safely and effectively achieve it. One possible way could be to use exosomes to deliver circRNAs without immunologic rejection. Exosomes are shown to contain stable circRNAs and can serve as diagnostic biomarkers for colon cancer detection [155]. A similar role of exosomes in RCC is yet to be revealed. It is evident that differential regulation of various circRNAs and their role in RCC development indicates their importance as potential therapeutic targets and biomarkers for the development of more effective treatment strategies for RCC therapy. However, there is still a need for more extensive research focused on circRNAs and their involvement in RCC.

## Abbreviations

circRNAs: Circular RNAs
RCC: Renal cell carcinoma
VHL: Von Hippel-Lindau
HIF: Hypoxia-inducible factors
EcircRNAs: Exonic circRNAs
IcircRNAs: Intronic circRNAs
EicircRNA: Exon–intron circRNAs
RNP: RNA binding protein
ccRCC: Clear cell renal cell carcinoma
EMT: Epithelial–mesenchymal transition
TME: Tumor microenvironment
